# Identification of a Novel Betacoronavirus (*Merbecovirus*) in Amur Hedgehogs from China

**DOI:** 10.3390/v11110980

**Published:** 2019-10-24

**Authors:** Susanna K. P. Lau, Hayes K. H. Luk, Antonio C. P. Wong, Rachel Y. Y. Fan, Carol S. F. Lam, Kenneth S. M. Li, Syed Shakeel Ahmed, Franklin W.N. Chow, Jian-Piao Cai, Xun Zhu, Jasper F. W. Chan, Terrence C. K. Lau, Kaiyuan Cao, Mengfeng Li, Patrick C. Y. Woo, Kwok-Yung Yuen

**Affiliations:** 1 Department of Microbiology, Li Ka Shing Faculty of Medicine, The University of Hong Kong, Hong Kong 999077, China; skplau@hku.hk (S.K.P.L.); hkhluk@hku.hk (H.K.H.L.); antonwcp@connect.hku.hk (A.C.P.W.); carollamsukfun@yahoo.com.hk (C.S.F.L.); keth105@gmail.com (K.S.M.L.); shakeel87@gmail.com (S.S.A.); caijuice@hku.hk (J.-P.C.); jfwchan@hku.hk (J.F.W.C.); 2 State Key Laboratory of Emerging Infectious Diseases, The University of Hong Kong, Hong Kong 999077, China; 3 Carol Yu Centre for Infection, The University of Hong Kong, Hong Kong 999077, China; 4 Collaborative Innovation Centre for Diagnosis and Treatment of Infectious Diseases, The University of Hong Kong, Hong Kong 999077, China; 5 Department of Microbiology, Zhongshan School of Medicine, Sun Yat-sen University, Guangzhou 510080, China; zhuxun8@mail.sysu.edu.cn (X.Z.); caoky@mail.sysu.edu.cn (K.C.); limf@mail.sysu.edu.cn (M.L.); 6 Key Laboratory of Tropical Disease Control (Sun Yat-sen University), Ministry of Education, Guangzhou 510080, China; 7 Department of Biomedical Sciences, Jockey Club College of Veterinary Medicine and Life Sciences, City University of Hong Kong, Hong Kong 999077, China; chiklau@cityu.edu.hk

**Keywords:** hedgehog, *Merbecovirus*, coronavirus, novel species, China

## Abstract

While dromedaries are the immediate animal source of Middle East Respiratory Syndrome (MERS) epidemic, viruses related to MERS coronavirus (MERS-CoV) have also been found in bats as well as hedgehogs. To elucidate the evolution of MERS-CoV-related viruses and their interspecies transmission pathway, samples were collected from different mammals in China. A novel coronavirus related to MERS-CoV, *Erinaceus amurensis* hedgehog coronavirus HKU31 (*Ea*-HedCoV HKU31), was identified from two Amur hedgehogs. Genome analysis supported that *Ea*-HedCoV HKU31 represents a novel species under *Merbecovirus*, being most closely related to *Erinaceus* CoV from European hedgehogs in Germany, with 79.6% genome sequence identity. Compared to other members of *Merbecovirus*, *Ea*-HedCoV HKU31 possessed unique non-structural proteins and putative cleavage sites at ORF1ab. Phylogenetic analysis showed that *Ea*-HedCoV HKU31 and BetaCoV Erinaceus/VMC/DEU/2012 were closely related to NeoCoV and BatCoV PREDICT from African bats in the spike region, suggesting that the latter bat viruses have arisen from recombination between CoVs from hedgehogs and bats. The predicted HKU31 receptor-binding domain (RBD) possessed only one out of 12 critical amino acid residues for binding to human dipeptidyl peptidase 4 (hDPP4), the MERS-CoV receptor. The structural modeling of the HKU31-RBD-hDPP4 binding interphase compared to that of MERS-CoV and *Tylonycteris* bat CoV HKU4 (*Ty*-BatCoV HKU4) suggested that HKU31-RBD is unlikely to bind to hDPP4. Our findings support that hedgehogs are an important reservoir of *Merbecovirus*, with evidence of recombination with viruses from bats. Further investigations in bats, hedgehogs and related animals are warranted to understand the evolution of MERS-CoV-related viruses.

## 1. Introduction

In 2012, the Middle East Respiratory Syndrome (MERS) first emerged and 27 countries have been affected with 2458 confirmed cases, together with a 34.5% fatality rate [1]. The etiological agent, MERS coronavirus (MERS-CoV), has been found in dromedaries from the Middle East and North Africa, which are generally believed to be the immediate animal source of the MERS epidemic [2,3,4]. While many related viruses have been discovered in other animals, namely bats and hedgehogs [5,6,7,8,9,10], the evolutionary path of MERS-CoV as well as its origin are yet to be ascertained. 

Coronaviruses (CoVs) are classified into four genera, *Alphacoronavirus*, *Betacoronavirus*, *Gammacoronavirus* and *Deltacoronavirus*, while *Betacoronavirus* is further subdivided into four lineages, A to D [11,12,13,14,15,16]. In 2018, these four *Betacoronavirus* lineages were reclassified into five subgenera, namely *Embecovirus, Sarbecovirus, Merbecovirus*, *Nobecovirus*, and an additional subgenus, *Hibecovirus* [17]. Notably, four of the six CoVs known to infect humans belong to *Betacoronavirus*, with human CoV HKU1 and OC43 belonging to *Embecovirus*, Severe Acute Respiratory Syndrome CoV (SARS-CoV) belonging to *Sarbecovirus* and MERS-CoV belonging to *Merbecovirus* [18,19,20,21,22,23,24,25]. The bloom in the discovery of novel animal CoVs since the SARS epidemic has uncovered bats as an important reservoir for alphacoronaviruses and betacoronaviruses, whereas birds are reservoirs responsible for gammacoronaviruses and deltacoronaviruses [14,26]. 

*Tylonycteris* bat CoV HKU4 (*Ty*-BatCoV HKU4) from Lesser bamboo bat (*Tylonycteris pachypus*) and *Pipistrellus* bat CoV HKU5 (*Pi*-BatCoV HKU5) from Japanese pipistrelle (*Pipistrellus abramus*) were discovered in Hong Kong and represent the first *Merbecovirus* (lineage C betacoronaviruses) discovered, five years before the outbreak of the MERS epidemic [6,15]. They were subsequently analyzed and the result suggested that they shared a close relationship with MERS-CoV, which raised the possibility that the animal origin of MERS-CoV belongs to bats [6,15,23,24,27,28]. A number of other *Merbecovirus* members were later discovered in bats, including Coronavirus BatCoV PREDICT/PDF-2180, Neoromicia/PML-PHE1/RSA/2011 (NeoCoV), *Hypsugo pulveratus* bat CoV HKU25 (*Hp*-BatCoV HKU25), and BtVs-BetaCoV/SC2013 from Africa and China [7,8,9,29]. Among these viruses, the spike proteins of *Ty*-BatCoV HKU4 and *Hp*-BatCoV HKU25 were able to bind and utilize human dipeptidyl peptidase 4 (hDPP4), the MERS-CoV receptor, for viral entry to the host cells, though with the highest efficiency in MERS-CoV, followed by *Ty*-BatCoV HKU4 and the lowest in *Hp*-BatCoV HKU25 [9,30]. The discovery of NeoCoV suggested it is the closest bat counterpart of MERS-CoV. Genomic analysis revealed that only the spike protein is genetically divergent from that of MERS-CoV throughout the whole genome [8], and hence is not likely that NeoCoV is the immediate ancestor of MERS-CoV. Besides bats, a member of *Merbecovirus*, Erinaceus CoV VMC/DEU (EriCoV), has been discovered in the fecal samples of European hedgehogs, *Erinaceus europaeus*, from Germany [5]. Similar viral sequences were also detected in European hedgehogs from France later [31], suggesting that mammals other than bats may contribute to the evolution of *Merbecovirus* including MERS-CoV. 

In order to explore the potential animal origin of MERS-CoV, as well as understanding the host diversity and evolutionary pathway of *Merbecovirus*, samples from various mammals were collected from China. We discovered a novel member of *Merbecovirus* from two Amur hedgehogs (*Erinaceus amurensis*). Genome analysis indicated that the virus belongs to a novel species under *Merbecovirus*, being most closely related to EriCoV from hedgehogs in Germany. The findings support that hedgehogs are an important reservoir of *Merbecovirus*. 

## 2. Materials and Methods 

### 2.1. Ethics Statement 

Hedgehog and rodent sample collection was approved by the Sun Yat-Sen University, Guangzhou, and the Committee on the Use of Live Animals for Teaching and Research, The University of Hong Kong (CULATR Ref. No.: 1486-07, 2284-10 and 3330-14; Date of approval: 26-04-2007, 23-03-2011 and 17-04-2014).

### 2.2. Sample Collection 

Hedgehogs, shrews and rodents were captured and sampled in Hong Kong and mainland China from January 2010 to August 2014. Alimentary samples were collected following the procedures described previously [32,33]. The Sun Yat-Sen University, Guangzhou was responsible for sample collection from mainland China. The Agriculture, Fisheries and Conservation Department (AFCD), and Food and Environmental Hygiene Department (FEHD) of the Hong Kong Special Administrative Region (HKSAR) were responsible for sample collection in Hong Kong. Viral transport medium (Earle’s balanced salt solution, 0.09% glucose, 0.03% sodium bicarbonate, 0.45% bovine serum albumin, 50 mg/mL amikacin, 50 mg/mL vancomycin, 40 U/mL nystatin) was used for transferring samples. A −80 °C freezer was used for sample storage before further processing. 

### 2.3. Detection of CoVs by RT-PCR and Sequencing 

Viral RNA from the samples were extracted following the suggested protocol of QIAamp Viral RNA Mini Kit (QIAgen, Hilden, Germany). A volume of 50 μL of eluted RNA was subjected to RT-PCR as the template. The partial region of RNA-dependent RNA polymerase (RdRp) gene (440 bp) of CoVs was targeted for viral detection using degenerated primers (5′-GGTTGGGACTATCCTAAGTGTGA-3′ and 5′-ACCATCATCNGANARDATCATNA-3′) [22]. Reverse transcription was carried out according to the suggested protocol of the SuperScript III kit (Invitrogen, Life Technologies, Grand Island, NY, USA). The amplification of 25 μL PCR mixtures was carried out in automated thermal cyclers (Applied Biosystems). Each reaction mixture contained PCR buffer (10 mM Tris-HCl pH 8.3, 3 mM MgCl_2_, 50 mM KCl and 0.01% gelatin), 200 μM of each dNTPs and 1.0 U *Taq* polymerase (Applied Biosystems, Life Technologies, Grand Island, NY, USA) together with sample cDNA. A total of 40 amplification cycles were set as 94 °C for 1 min, 48 °C for 1 min and 72 °C for 1 min, followed by a 10 min final extension at 72 °C. Each run included negative controls so as to avoid a false-positive result and PCR contamination.

Amplified PCR products were examined by gel electrophoresis. Targeted products were purified and sequenced using the QIAquick gel extraction kit (QIAgen) and an ABI Prism 3700 DNA Analyzer (Applied Biosystems), respectively. A comparison between obtained viral sequences with known CoVs’ sequences from the GenBank database was performed. The 383 bp fragments of RdRp genes were subjected to phylogenetic tree construction. The maximum likelihood method and General Time Reversible model were applied with Gamma Distribution and an allowance of evolutionarily invariable sites (GTR+G+I) in the analysis using PhyML v3.0 (The French Institute of Bioinformatics & France Genomique, Montpellier, France) [28,34,35]. 

### 2.4. Viral Culture 

Various cell lines were used to perform the viral isolation of the two positive samples for *Ea*-HedCoV HKU31, including Vero E6 (African green monkey kidney) (no. CRL-1586, American Type Culture Collection, Manassas, VA, USA), PK15 (porcine kidney) (no. CCL-33, American Type Culture Collection), *Pipistrellus abramus*-immortalized lung and kidney cells, *Hipposideros pomonas*-immortalized brain and kidney cells and *Rousettus lechenaultii*-immortalized kidney cells [36,37]. A volume of 200 μL of each sample was added to the cells and incubated at 37 °C for 1 h. The inoculum was discarded, and the cells were washed with PBS twice before the addition of fresh culture medium with antibiotics. The cells were then incubated at 37 °C for 5 days in each passage. Three blind passages were carried out for each positive sample. A daily observation for cytopathic effect was performed. Both cell culture supernatant and cell pellet were collected in day 5 of post infection and subjected to RNA extraction followed by RT-PCR for viral detection. 

### 2.5. Complete Genome Sequencing and Analysis of Ea-HedCoV HKU31 

Complete genomes of two *Ea*-HedCoV HKU31 strains were amplified by PCR and sequenced from fecal and rectal swab samples. Combined random-priming together with oligo(dT) priming strategy were used to convert RNA to cDNA, followed by amplification using degenerate primers [32]. A total of 72 sets of primers were designed and used for complete genome sequencing (available on request). Rapid amplification of cDNA ends was conducted to obtain the 5′end viral genome using the 5′/3′ SMARTer^TM^ RACE cDNA Amplification Kit (Clontech, Mountain View, CA, USA). Final genomic sequences were obtained by manual editing and assembly. Both nt and aa genomic sequences were compared to other CoVs obtained from GenBank. Phylogenetic tree, with 1000 bootstrap values calculation, was constructed using the maximum likelihood method using PhyML v3.0 (Montpellier, France) together with the best-fit substitution models selected by Smart Model Selection. PFAM and InterProScan were used to analyze protein family while transmembrane domains prediction was done by TMHMM [38,39,40]. 

### 2.6. Estimation of Divergence Dates 

Estimation of the most recent common ancestor (tMRCA) was conducted using the uncorrelated exponential distributed relaxed clock model (UCED) selected from BEAST version 1.8.3 (http://evolve.zoo.ox.ac.uk/beast/) [41], in which the rates vary at each branch drawn independently from an exponential distribution. The sampling dates, obtained from literatures or this study, were included as calibration points. The nt substitution model (GTR + G + I) was applied in Markov chain Monte Carlo (MCMC) run. In total, 2 × 10^8^ steps along with sampling every 1000 steps were used. The highest posterior density regions at 95% (HPDs) together with the mean time to tMRCA were analyzed and calculated. A 10% burn-in using TRACER v1.6 was performed and followed by assessing the convergence on the basis of the effect sample size. Annotation of the tree was performed by TreeAnnotator program included in the BEAST package and the finalized tree was displayed by FigTree (http://tree.bio.ed.ac.uk/software/figtree/).

### 2.7. Structural Modelling of Ea-HedCoV HKU31 Receptor-Binding Domain (RBD) 

The HKU31-RBD and HKU4-RBD models were built with the MERS-RBD/human CD26 complex (4KR0) crystal structure using SWISS-MODEL based on default parameters. To ensure the residues of the structure were not located in unfavorable region, the Ramachandran plot of each model was examined. The interacting residues of RBD in the models were analyzed and highlighted using Discovery Studio visualizer (Accelrys, San Diego, USA). The model of MERS-CoV RBD was built as a positive control.

### 2.8. Recombination Analysis 

Possible recombination was detected using bootscan analysis with software Simplot version 3.5.1 (SCRoftware, US). The nucleotide genome sequences of selected Merbecoviruses were aligned and further analyzed with 1000 bootstrap replicates. The sliding window was set with 1000 nucleotides together with 200 nucleotides moving steps. 

### 2.9. Nucleotide Sequence Accession Numbers 

The genome sequences of *Ea*-HedCoV HKU31 have been lodged within the GenBank sequence database under accession no. MK907286-MK907287.

## 3. Results

### 3.1. Detection of CoVs in Animals and Discovery of a Novel Species of Merbecovirus from Amur Hedgehogs 

**Figure 1:**
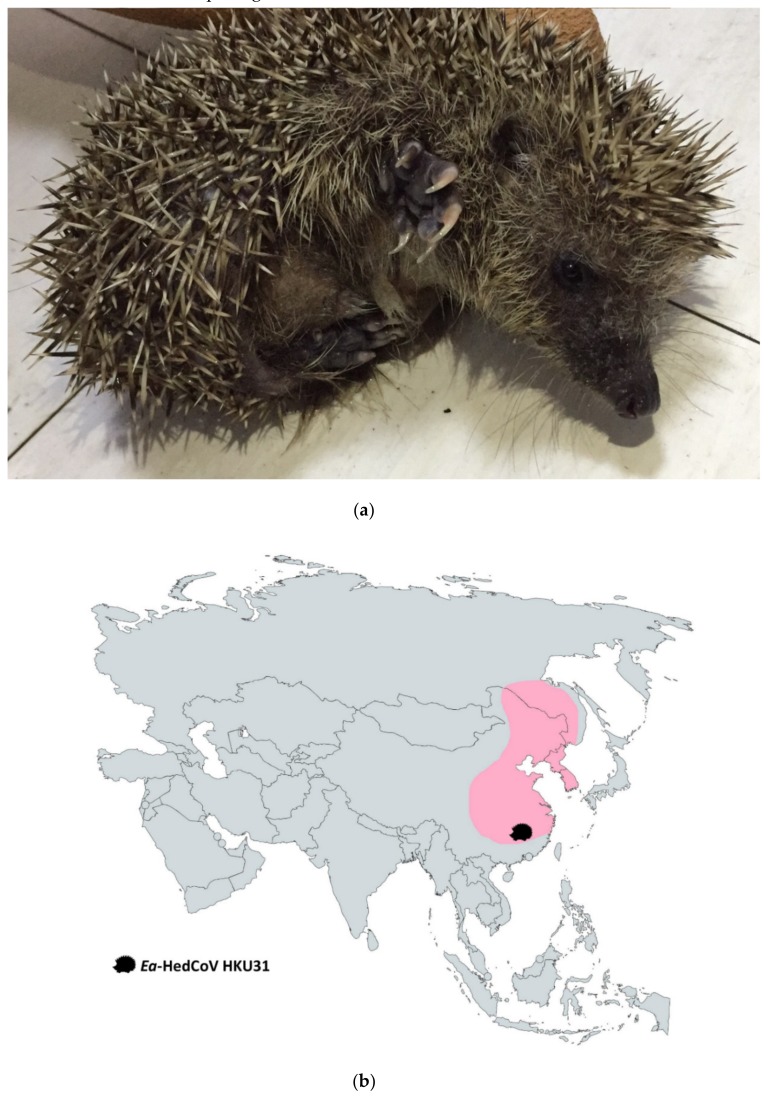
(**a**) *Erinaceus amurensis*, also known as Amur hedgehog or Manchurian hedgehog, sampled in this study; (**b**) Geographical distribution of *Erinaceus amurensis* in Asia. The colored region represents the habitat where *Erinaceus amurensis* resides. The labeled area represents the location where Ea-HedCoV HKU31 was discovered.

A total number of 207 alimentary samples, 27 from hedgehogs, 151 from rodents and 29 from shrews were obtained from various regions of the Guangdong province of China and Hong Kong. Reverse-transcription polymerase chain reaction (RT-PCR) for a partial RNA-dependent RNA polymerase (RdRp) gene fragment (440bp) and sequencing showed the presence of *Merbecovirus* in two samples from two Amur hedgehogs (Erinaceus amurensis) (Figure 1a,b and Table S1). Sequence analysis suggested a potentially novel species in *Merbecovirus* was found from two samples (F6 and RS13) (Figure S1), which shared 86% nt identity to Betacoronavirus Erinaceus/VMC/DEU/2012, 84% nt identity to Betacoronavirus Eptesicus/13RS384_26/Italy/2012 and 85–86% nt identity to MERS-CoV. We proposed Erinaceus amurensis hedgehog coronavirus HKU31 (*Ea*-HedCoV HKU31) as the name of this novel CoV. Attempts to passage *Ea*-HedCoV HKU31 F6 and RS13 in different cell cultures were not successful including Vero, PK15, *Pipistrellus abramus*-immortalized lung and kidney cells, *Hipposideros pomonas*-immortalized brain and kidney cells and *Rousettus lechenaultii*-immortalized kidney cells. There was no cytopathic effect observed or detectable viral RNA by RT-PCR for both cell culture supernatants and lysates after 5 days of incubation in all three blind passages. 

### 3.2. Genome Organization of Ea-HedCoV HKU31 

To determine the evolutionary relationship between *Ea*-HedCoV HKU31 and MERS-CoV, two complete genome sequences of *Ea*-HedCoV HKU31 strains, F6 and RS13, were determined by assembly of RT-PCR products sequences obtained from the alimentary samples. Both viral strains contained 29951 to 29955 bases in genome sizes, with G + C content 37.7% (Table 1). They shared 99.8% overall nt identities, while possessing 79.6%, 68.4% and 69.2% nt identities to the genomes of Erinaceus CoV/2012-174.GER.2012, human/camel MERS-CoVs and NeoCoV respectively. Comparison of the seven conserved replicase domains, nsp3 (ADRP), nsp5 (3CL^pro^), nsp12 (RdRp), nsp13 (Hel), nsp14 (ExoN), nsp15 (NendoU) and nsp16 (O-MT), for CoV species demarcation showed that *Ea*-HedCoV HKU31 possessed 53.1%–71.1%, 71.2%–85.9%, 87.6%–91.3%, 89.8%–95.7%, 85.1%–92.1%, 82.2%–90.7% and 82.8%–90.4% aa identities to other members of *Merbecovirus* respectively (Table S2). The results support that *Ea*-HedCoV HKU31 represents a novel species under *Merbecovirus*.

**Figure 2:**
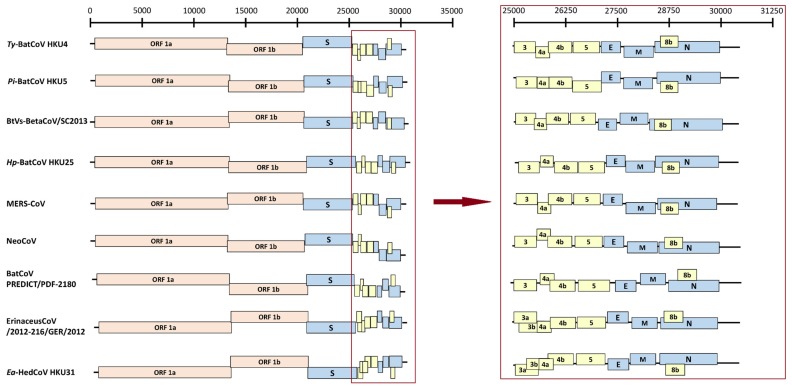
Comparison of genome organizations of *Ea*-HedCoV HKU31 with other members of *Merbecovirus*, including MERS-CoV, BtVs-BetaCoV SC2013, *Ty*-BatCoV HKU4, *Pi*-BatCoV HKU5, *Hp*-BatCoV HKU25, BatCoV PREDICT/PDF-2180, Erinaceus CoV/2012-216/GER/2012 and NeoCoV. Structural proteins such as spike (S), envelope (E), membrane (M) and nucleocapsid (N) are displayed in blue boxes, while accessory proteins are displayed in yellow boxes with numbering 3-5 and 8b. ORF1a and ORF1b are represented by pink boxes.

The genome organization of *Ea*-HedCoV HKU31 was similar to other members of *Merbecovirus* (Figure 2). A putative transcription regulatory sequence (TRS) motif, 5’-AACGAAC-3’, typical of Betacoronavirus (except Embecovirus), was identified at the 3’ end of leader sequence and preceded each ORF except N with an alternative motif, 5’-AACGAAU-3’. Predicted functional domains in the different ORFs are summarized in Table S3. The ORF1ab polyprotein possessed 43.6%–81.8% aa identities to the polyproteins of other members of *Merbecovirus*. The lengths of nsp1, nsp2, nsp3, nsp10, nsp15 and nsp16 in *Ea*-HedCoV HKU31, as predicted by putative cleavage sites, were different from those in other members of *Merbecovirus*, as a result of deletions or insertions (Table S4). 

### 3.3. Sequence Analysis and Structural Modelling of the Ea-HedCoV HKU31 Spike Protein

Similar to other CoVs, the prediction of *Ea*-HedCoV HKU31 S suggests that it belongs to type I membrane glycoprotein, with a large portion of the glycoprotein (residues 21–1270) exposed on the outside and one transmembrane domain (residues 1271–1294) at the C terminus. One heptad repeat (HR), which is responsible for membrane fusion together with viral entry, was found from residues 1219 to 1257 (HR2). *Ea*-HedCoV HKU31 S possessed 77.7%, 57.5% and 57.2% aa identities to the S of Erinaceus CoV/VMC/DEU/2012, MERS-CoV and *Ty*-BatCoV HKU4 respectively. Moreover, the predicted *Ea*-HedCoV HKU31 S1-RBD shared 40.8% aa identities to MERS-CoV S1-RBD, with three deletions of 1, 3 and 4 aa respectively (Figure S2). 

**Figure 3:**
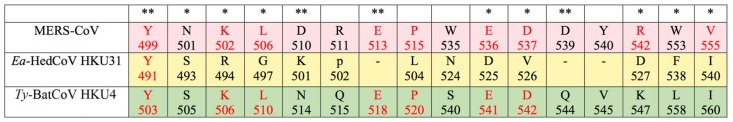
Multiple sequence alignment showing variations in key amino acid binding residues. Conserved residues are highlighted in red. Critical residues and critical bond formation residues are labelled with (*)/(**) respectively. Pink boxes refer to amino acid residues of MERS-CoV. Yellow boxes refer to amino acid residues of *Ea*-HedCoV HKU31. Green boxes refer to amino acid residues of *Ty*-BatCoV HKU4.

**Figure 4:**
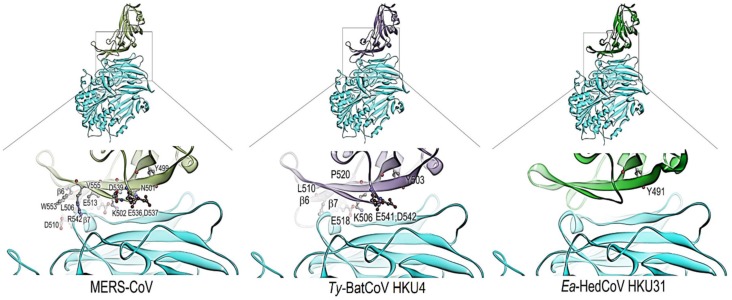
The models of receptor-binding domains (RBDs) of MERS (pea green), HKU4-4 S (purple) and HKU31-F6 S (green) are shown with hDPP4 structure (light blue) in ribbon diagram. The interface of different RBDs and hDPP4 are zoomed into and the residues that were highlighted in multiple sequence alignment from Figure 5 are shown in ball-and-stick format, colored by element (carbon, gray; nitrogen, blue; oxygen, red). Strands of β6 and β7 are present in the structure of MERS and HKU4-4S only. The figures were produced using Discovery Studio visualizer (Accelrys).

A type II transmembrane protein, hDPP4, was utilized by MERS-CoV as the receptor for initiation of infection, with a 240-residue RBD at C-terminal of S1 domain being responsible for receptor binding [42]. 12 critical residues, including Y499, N501, K502, L506, D510, E513, E536, D537, D539, R542, W553 and V555 were identified in previous studies for hDPP4 and MERS-CoV RBD binding [42,43]. *Ty*-BatCoV HKU4 also utilizes hDPP4 receptor during cell entry. Its spike protein possessed five to seven (Y503, K506, L510, E518, P520, E541, D542) of the 12 critical residues [44]. In *Ea*-HedCoV HKU31, only one residue (Y491) was conserved in both strains (Figure 3). This residue, corresponding to Y499 in MERS-CoV, is part of the hydrogen bond formation between MERS-CoV RBD and hDPP4 that plays an important role in terms of viral-host receptor binding and viral entry into the cells [42,43]. The prediction of the RBD-hDPP4 binding-interphase was carried out. The structures of HKU31-, HKU4- and MERS-RBDs were built and modelled with the structure of hDPP4 using homology modelling. HKU31-RBD and MERS-RBD (template) shared <50% (40.3%) sequence identity. As shown in Figure 4, only HKU4-RBD and MERS-RBD but not HKU31-RBD possessed the extended loop located in between β6 and β7, which have been shown to play a role in the interaction of hDPP4 [44]. This suggests that HKU31-RBD is likely unable to bind to hDPP4. 

### 3.4. Phylogenetic Analysis 

**Figure 5:**
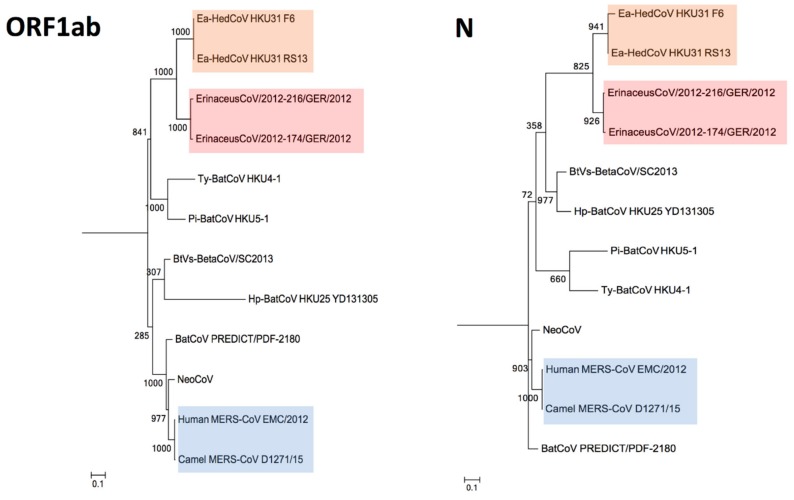
Phylogenetic analyses of ORF1ab and N amino acid sequences of *Ea*-HedCoV HKU31 and other members of *Merbecovirus*. The maximum likelihood method was used to construct ORF1ab and N by using the LG+G+F substitution model. Bootstrap values were calculated from 1000 trees. Corresponding sequences of HCoV HKU1 were included for trees rooting (GenBank accession number NC_006577). All bootstrap values are shown. The scale bars represent 10 substitutions per site in ORF1ab and N trees respectively. Human and camel MERS-CoVs are highlighted in blue. The two Erinaceus betacoronaviruses from Europe are highlighted in red. The two *Ea*-HedCoV HKU31 strains, F6 and RS13, detected in this study are highlighted in yellow.

**Figure 6:**
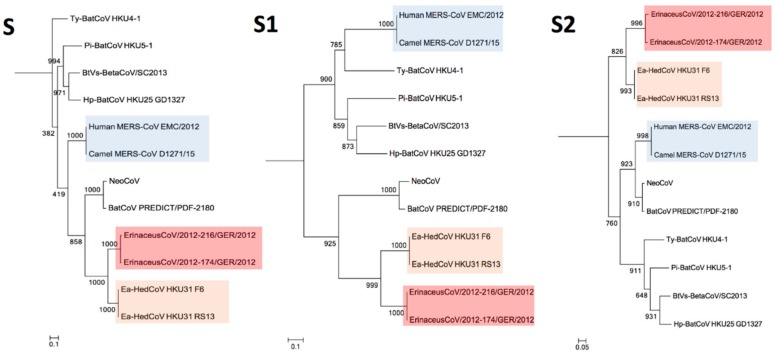
Phylogenetic analyses of S, S1 and S2 amino acid sequences of *Ea*-HedCoV HKU31 and other members of *Merbecovirus*. The maximum likelihood method was used to construct S and S1 tree by using WAG+G+F substitution models. The maximum likelihood method was used to construct S2 tree by using LG+G+F substitution model. Bootstrap values were calculated from 1000 trees. Corresponding sequences of HCoV HKU1 were included for trees rooting (GenBank accession number NC_006577). All bootstrap values are shown. The scale bars represent 10, 10 and 20 substitutions per site in S, S1 and S2 trees respectively. Human and camel MERS-CoVs are highlighted in blue. The two Erinaceus betacoronaviruses from Europe are highlighted in red. The two *Ea*-HedCoV HKU31 strains, F6 and RS13, detected in this study are highlighted in yellow.

The construction of phylogenetic trees using ORF1ab, S, S1, S2 together with N sequences of *Ea*-HedCoV HKU31 are performed and shown as Figure 5 and Figure 6. In all the trees, *Ea*-HedCoV HKU31 was found to be most closely related to BetaCoV Erinaceus/VMC/DEU/2012, forming a distinct branch among *Merbecovirus*. Notably, in S and especially in the S1 region, this distinct “hedgehog” branch was most closely related to NeoCoV and BatCoV PREDICT from Africa, forming a separate clade from other members of *Merbecovirus*. However, in the ORF1ab and N regions, this “hedgehog” branch was more closely related to other bat viruses detected previously in China [29], while NeoCoV and BatCoV PREDICT were most closely related to MERS-CoVs. 

### 3.5. Estimation of Divergence Dates

The time of the most recent common ancestor (tMRCA) of *Ea*-HedCoV HKU31 and BetaCoV Erinaceus/VMC/DEU/2012 was estimated using the uncorrelated relaxed clock model on ORF1ab and it was found to be approximately 1913 [highest posterior density regions at 95% (HPD), 616 to 2008] (Figure S3). The tMRCA of all members of *Merbecovirus* were estimated at approximately 1580 [highest posterior density regions at 95% (HPD), 4025 BC to 1976].

### 3.6. Recombination Analysis

**Figure 7:**
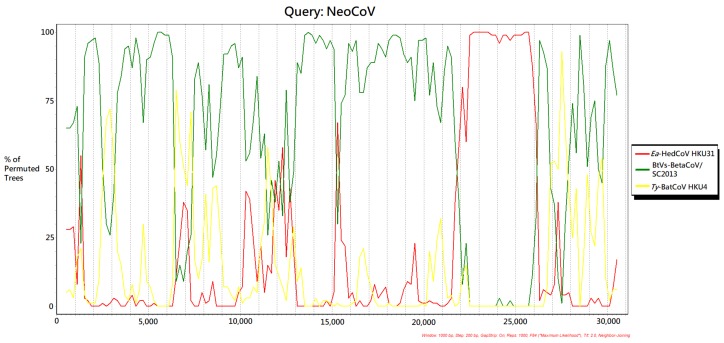
Detection of a potential recombination event by bootscan analysis. Simplot version 3.5.1 was used to perform the analysis with the F84 model, with a 1000 bp window size and 200 bp moving step. NeoCoV was chosen as the query sequence and compared with other Merbecoviruses including *Ea*-HedCoV HKU31 (red), BtVs-BetaCoV/SC2013 (green) and *Ty*-BatCoV HKU4 (yellow).

The NeoCoV genome showed different clustering positions in ORF1ab, S (especially in S1 region) and N phylogenetic trees (Figure 5 and Figure 6). Possible recombination between NeoCoV and other Merbecoviruses was suggested and it was subjected to recombination analysis. Using NeoCoV as query for bootscan analysis, a possible recombination site was revealed at the aligned genome position starting from approximately 21,700 to 26,100, which shared a closer relationship with *Ea*-HedCoV HKU31 (Figure 7). For the rest of the genome regions, NeoCoV showed closer relationships with other bat CoVs BtVs-BetaCoV/SC2013 or *Ty*-BatCoV HKU4 (Figure 7). 

## 4. Discussion

We discovered a novel species of *Merbecovirus*, *Ea*-HedCoV HKU31, in Amur hedgehogs from China. The genome of *Ea*-BatCoV HKU31 was most closely related to that of Betacoronavirus Erinaceus/VMC/DEU/2012, with 79.6% nt identities, and shared 68.4% nt identities to that of MERS-CoV. It contains additional non-structural proteins that are different from those of other members of *Merbecovirus*. Phylogenetically, *Ea*-HedCoV HKU31 and Betacoronavirus Erinaceus/VMC/DEU/2012 represent a distinct clade among *Merbecovirus*. However, this “hedgehog” branch was closely related to NeoCoV and BatCoV PREDICT from African bats in the spike region. Interestingly, NeoCoV is known to be the closest bat CoV to MERS-CoV, sharing high genomic similarity in most regions except the spike gene [7]. On the other hand, *Ty*-BatCoV HKU4 possesses a spike gene being the closest to that of MERS-CoV. It was shown that the presence of hDPP4 could allow *Ty*-BatCoV HKU4 to enter targeted cells [44]. A novel species of batCoV in *Merbecovirus*, *Hp*-BatCoV HKU25, which was recently reported by our group, with a spike protein also capable of binding and utilizing hDPP4 for cell entry but with a lower efficiency than that of *Ty*-BatCoV HKU4 [9]. CoVs are known for their tendency for recombination, which may facilitate the emergence of novel viruses that may infect new hosts, as best illustrated with the SARS epidemic [32,45,46,47,48,49]. Previous studies suggested that MERS-CoV may be a recombinant virus between NeoCoV and other bat CoVs [7]. However, results from the present study supported our previous hypothesis that NeoCoV and related viruses were from a recombinant virus between hedgehog CoVs and bat CoVs in *Merbecovirus* [9]. This is also consistent with a recent report of a novel bat CoV discovered in a bat species, *Ia io*, with natural recombination with *Ty*-BatCoV HKU4 [10]. The present findings provide further evidence that hedgehogs from different geographical areas are natural reservoir of *Merbecovirus*. 

While hedgehogs are unlikely to be the animal origin of MERS-CoV, these hedgehog viruses may recombine with bat viruses to generate new viruses in bats with potential of interspecies transmission. The spike protein of *Ea*-BatCoV HKU31 contained three deletions in the predicted S1-RBD when compared to the MERS-CoV spike protein, with only one of the 12 critical residues being conserved for MERS-RBD to hDPP4 binding. Structural modelling also predicted low homology between MERS-RBD and HKU31-RBD in the RBD-hDPP4 interphase, suggesting that the *Ea*-BatCoV HKU31 spike protein may not be able to bind and utilize hDPP4 as receptor. However, the potential of these hedgehog viruses with cross-species barrier ability remains to be elucidated as the receptor utilized by this virus remains unknown. Pseudovirus assays with different host receptors may allow scientists to further investigate the cell entry mechanism of these hedgehog viruses. This notwithstanding, they are potential for recombination leading to new viruses as in the case of NeoCoV. According to other molecular dating studies performed previously, the divergence time of MERS-CoVs was estimated as 2009 to 2011 approximately [34,50,51,52,53]. The dating results in this study are in line with the previous estimations, with the tMRCA of MERS-CoVs dated to approximately 2009, that of hedgehog CoVs dated to approximately 1913 and that of all members of *Merbecovirus* dated to approximately 1580. This indicates that the hedgehog viruses may have only emerged a century ago and the recombinant ancestor of NeoCoV and related viruses no earlier. Further evolutionary studies on *Merbecovirus* may help understand this rapidly evolving group of CoVs with emergence potential. 

Bats and hedgehogs in the Middle East, Africa and other regions should be continuously surveilled, which may further help delineate the animal origin of MERS-CoV. Comparatively, SARSr-CoVs are mainly found in horseshoe bats of the family *Rhinolophidae* while *Merbecovirus* was observed to infect a diverse host range in bats and hedgehogs. Previously reported batCoVs in *Merbecovirus* have been found in various genera of the bat family *Vespertilionidae* [7,8,29,54,55,56,57,58]. Specifically, *Ty*-BatCoV HKU4, *Pi*-BatCoV HKU5, *Hp*-BatCoV HKU25 and BtVs-BetaCoV/SC2013 were detected in bats belonging to the genera, *Tylonycteris*, *Pipistrellus*, *Hypsugo* and *Verspetilio*, respectively, whereas NeoCoV and BatCoV PREDICT/PDF-2180 were detected in bats belonging to *Neoromicia* and *Pipistrellus* in Africa, respectively [7,8]. As for hedgehogs, *Merbecovirus* have only been detected in the genus *Erinaceus* in both Europe and China. Amur hedgehogs are found in various provinces of China and Korea. Interestingly, bats and hedgehogs are phylogenetically closely related and are both insectivorous animals. Further studies on insectivorous mammals of the order *Eulipotyphla* that are related to bats, such as hedgehogs and related animals, moles, solenodons and shrews, may further shed light on the evolution and diversity of *Merbecovirus*.

## Figures and Tables

**Table 1:** Genomic features of *Ea*-HedCoV HKU31 and other members of *Merbecovirus* which complete genome sequences are available and amino acid identities between the predicted proteins of *Ea*-HedCoV HKU31 and the corresponding proteins of other members of *Merbecovirus*.

	Genome Features		Pairwise aa Sequence Identity with *Ea*-HedCoV HKU31 Strain F6 Sequence (%)						
Coronavirus	Size (No. of Bases)	G+C Content	3CLPro	RdRp	Hel	S	E	M	N
*Merbecovirus*									
*Hp*-BatCoV HKU25	30,497	0.42	79.4	90.0	92.5	57.1	76.8	78.4	67.2
BtVs-BetaCoV/SC2013	30,423	0.43	78.4	90.7	92.6	57.3	79.3	79.8	68.9
Human MERS-CoV ChinaGD01	30,114	0.41	77.8	89.4	91.3	57.5	73.2	79	66.7
Human MERS-CoV England1	30,111	0.41	77.8	89.3	91.3	57.7	73.2	78.5	67.2
Human MERS-CoV EMC/2012	30,119	0.41	77.8	89.4	91.1	57.7	73.2	79	67.2
Camel MERS-CoV NRCE-HKU205	29,908	0.41	78.1	89.4	91.1	57.8	73.2	79	66.4
Camel MERS-CoV Jeddah-1	29,851	0.41	77.8	89.4	91.3	57.7	73.2	79	67.2
NeoCoV	30,111	0.40	77.1	89.2	91.6	63.4	75.6	80.4	69.7
*Ty*-BatCoV HKU4	30,286	0.38	71.2	87.6	90.8	57.5	64.6	77.6	63.8
*Pi*-BatCoV HKU5	30,488	0.43	75.5	89.1	91	57.7	62.2	79.1	63.8
BetaCoV/Erinaceus/VMC/DEU	30,175	0.37	85.6	92.2	96.2	77.9	84.1	90.8	84.8
*Ea*-HedCoV HKU31 RS13	29,951	0.38	100	100	100	99.9	100	100	96.5

## Supplementary Materials

The following are available online at https://www.mdpi.com/1999-4915/11/11/980/s1: Figure S1: Phylogenetic analysis of the nt sequences of the 383-nt fragment of RdRp of the two positive samples identified in hedgehogs from China in this study, Figure S2: Multiple alignment of the amino acid sequences of the receptor-binding domain (RBD) of the spike protein of MERS-CoV and corresponding sequences in *Ea*-HedCoV HKU31 and other members of *Merbecovirus*. Asterisks indicate positions with fully conserved residues. The three amino acid deletions in *Ea*-HedCoV HKU31 compared to MERS-CoV and *Ty*-BatCoV HKU4 are highlighted in red color, Figure S3: Estimation of tMRCA of members of *Merbecovirus* based on Orf1ab. The mean estimated dates were labeled. The taxa were labeled with their sampling dates, Table S1: Detection of CoVs in different hedgehog and rodent species by RT-PCR of the 440-bp fragment of RdRp gene, Table S2: Pairwise comparisons of *Coronaviridae*-wide conserved domains in replicase polyprotein 1ab between *Ea*-HedCoV HKU31 and other members of *Merbecovirus*, Table S3: Coding potential and predicted domains in different proteins of *Ea*-HedCoV HKU31, Table S4: Cleavage site used between nsps in members of *Merbecovirus.*

## Abbreviations

BetaCoV	Betacoronavirus
bp	Base pair
CoVs	Coronaviruses
HCoV	Human coronavirus
MERS-CoV	Middle East respiratory syndrome coronavirus
ORF	Open reading frame
PCR	Polymerase chain reaction
Pi	Pipistrellus
RdRp	RNA-dependent RNA polymerase
RT	Reverse transcription
S	Spike
SARSr-CoV	Severe Acute Respiratory Syndrome-related coronavirus
TRS	Transcription regulatory sequence
Ty	Tylonycteris
µL	Microliter
