# Supplementary material for: Identification of a Novel Betacoronavirus (Merbecovirus) in Amur Hedgehogs from China
Source: Viruses. 2019 Oct 24;11(11):980. doi: 10.3390/v11110980 (PMC6893546; doi:10.3390/v11110980)
Supplement: Supplementary file 1 [file viruses-11-00980-s001.pdf]

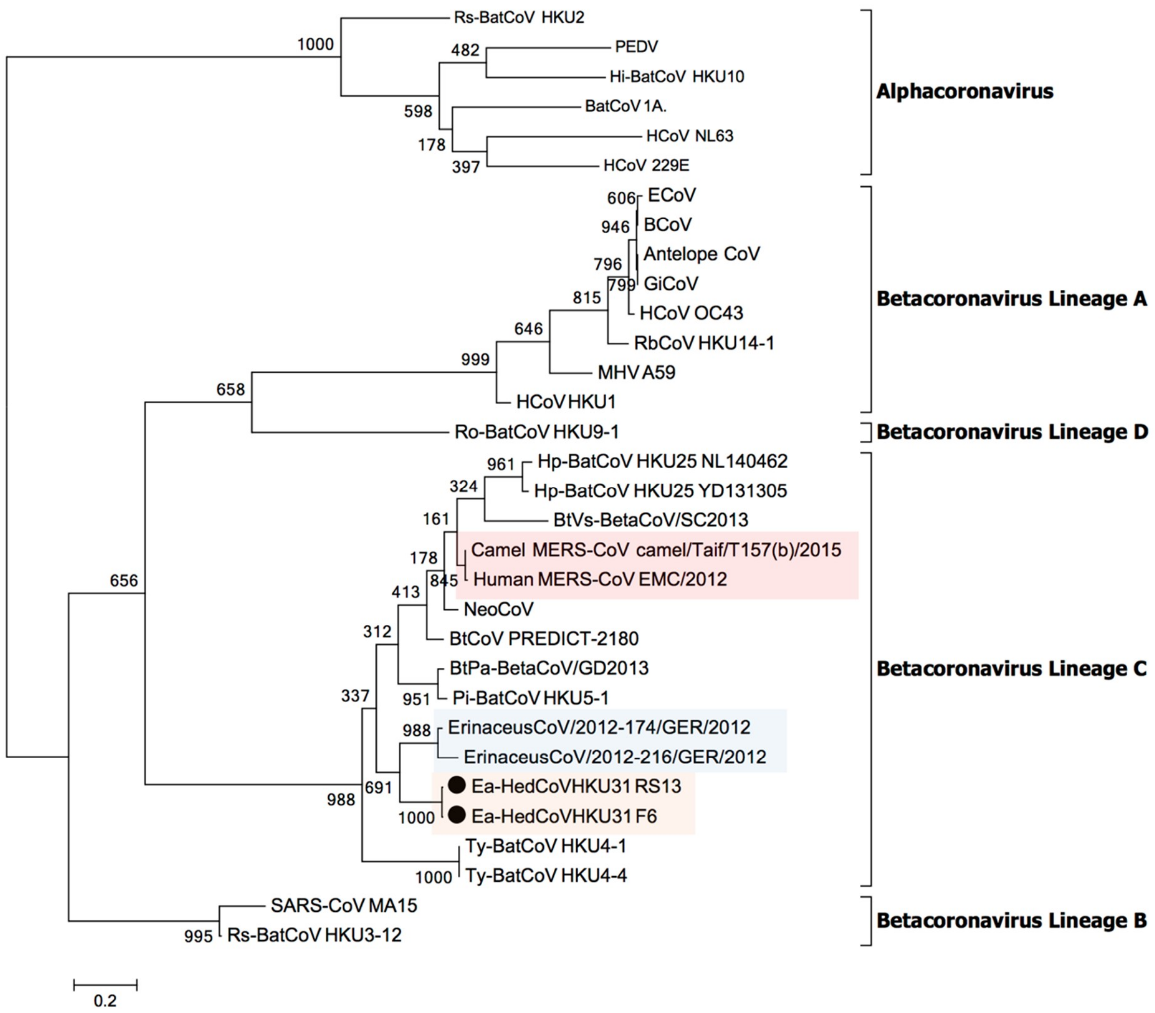

Figure S1. *Phylogenetic analysis of the nt sequences of the 383-nt fragment of RdRp of the 2 positive samples identified in hedgehogs from China in this study.*

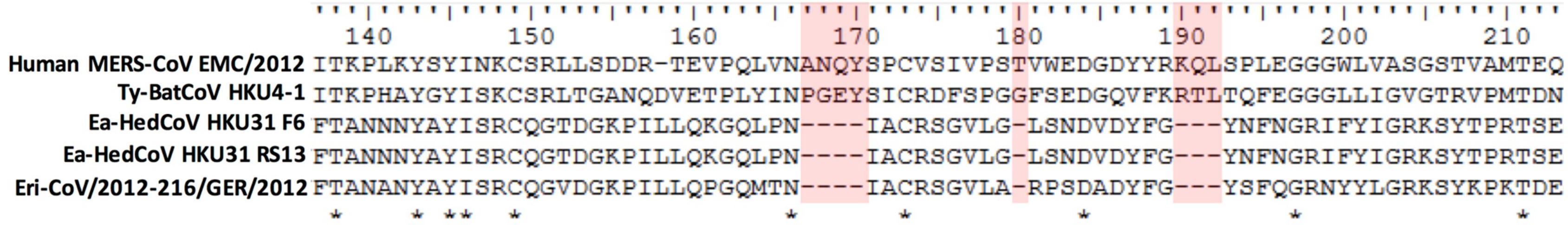

Figure S2. *Multiple alignment of the amino acid sequences of the receptor-binding domain (RBD) of the spike protein of MERS-CoV and corresponding sequences in Ea-HedCoV HKU31 and other members of Merbecovirus. Asterisks indicate positions with fully conserved residues. The three amino acid deletions in Ea-HedCoV HKU31 compared to MERS-CoV and Ty-BatCoV HKU4 are highlighted in red color.*

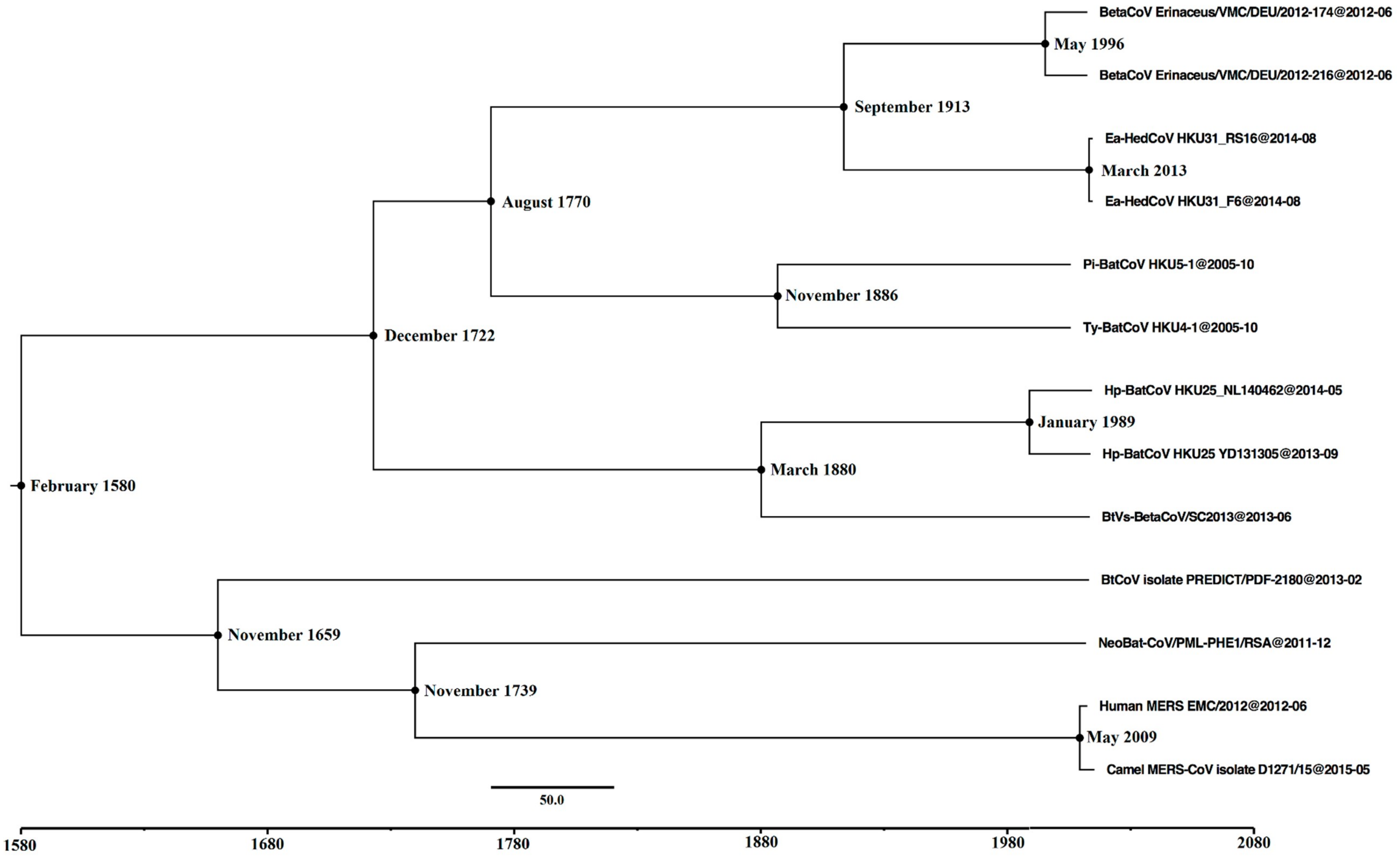

Figure S3. *Estimation of tMRCA of members of Merbecovirus based on Orf1ab. The mean estimated dates were labeled. The taxa were labeled with their sampling dates.*

Table S1. *Detection of CoVs in different hedgehog and rodent species by RT-PCR of the 440-bp fragment of RdRp gene*

Scientific name	Common name	No. of samples tested	No. of samples positive for CoV	CoV detected/closest match in GenBank	nt identity to closest match (%)	Sampling location of samples
Suncus Murinus	Asian house shrew	18	0/18 (0)	NA	N/A	Hong Kong
Crocidura attenuata	Asian gray shrew	11	0/11 (0)	NA	N/A	Hong Kong
Erinaceus amurensis	Amur hedgehog	27	2/27 (7.4)	Betacoronavirus Erinaceus/VMC/DEU/2012	84	Guangdong, China
Rattus rattus	Black rat	35	0/35 (0)	NA	N/A	Guangdong, China
Rattus andamanensis	Indochinese forest rat	116	0/116 (0)	NA	N/A	Guangdong, China

Table S2. *Pairwise comparisons of Coronaviridae-wide conserved domains in replicase polyprotein 1ab between Ea-HedCoV HKU31 and other members of Merbecovirus*

**Replicase polyprotein domain**	**Pairwise amino acid sequence identity with the *Ea*-HedCoV HKU31 sequence (%)**						
	**Erinaceus CoV**	**MERS-CoV**	**NeoCoV**	**SC2013**	***Ty*-BatCoV HKU4**	***Pi*-BatCoV HKU5**	***Hp*-BatCoV HKU25**
nsp3 (ADRP)	71.1	59.7	59.5	58.7	53.4	53.1	57.5
nsp5 ($3CL^{pro}$)	85.9	77.8	77.1	78.4	71.2	75.5	78.8
nsp12 (RdRp)	91.3	89.4	89.2	90.7	87.6	89.1	90.3
nsp13 (Hel)	95.7	91.1	91.6	91.6	89.8	90.5	91.8
nsp14 (ExoN)	92.1	88.7	88.9	89.3	85.1	88.7	88.5
nsp15(NendoU)	90.7	82.5	82.2	83.1	74.6	76.6	83.4
nsp16 (O-MT)	90.4	87.8	87.8	86.8	82.8	83.8	87.8
Concatenated domains	83.5	76.7	76.6	75.5	72.0	72.9	79.3

Table S3. *Coding potential and predicted domains in different proteins of Ea-HedCoV HKU31*

ORF	Nucleotide position (start-end)	No. of nucleotides	No. of amino acids	Frame(s)	Putative function of domain[a]	Amino acid positions	Putative TRS	
							Nucleotide position in genome	TRS sequence (distance [no. of bases] to AUG)[b]
1ab	244-21497	21254	7020	+1,+3			61	**AACGAAC** (176)AUG
nsp1	244-843	600	200	+1	Unknown	1-200		
nsp2	844-2820	1977	659	+1	Unknown	201-859		
nsp3	2821-8490	5670	1890	+1	ADRP,<br>putative domain PL1^{pro}<br>Nucleic Acid Binding domain<br>Single stranded polyA binding domain	860-2749		
nsp4	8491-10005	1515	505	+1	Hydrophobic domain	2750-3254		
nsp5	10006-10923	918	306	+1	3CL^{pro}	3255-3560		
nsp6	10924-11799	876	292	+1	Hydrophobic domain	3561-3852		
nsp7	11800-12048	249	83	+1	Peptidase	3853-3935		
nsp8	12049-12645	597	199	+1	Peptidase	3936-4134		
nsp9	12646-12975	330	110	+1	RNA binding domain	4135-4244		
nsp10	12976-13392	417	139	+1	RNA binding domain<br>Zinc ion binding motif	4245-4383		
nsp12	13393-16194	2802	934	+3	RdRp	4384-5317		
nsp13	16195-17988	1794	598	+3	Hel	5318-5915		
nsp14	17989-19560	1572	524	+3	ExoN, N7-MTase	5916-6439		
nsp15	19561-20589	1029	343	+3	NendoU	6440-6782		
nsp16	20590-21495	906	302	+3	O-MT	6783-7084		
S	21442-25425	3984	1327	+1	Type 1 membrane glycoprotein		21390	**AACGAAC**(45)AUG
					Receptor binding domain	368-621		
					Cleavage site	760/761		
					Two heptad repeats	984-1097 (HR1),<br>1219-1257 (HR2)		
					Transmembrane domain	1289-1311		
ORF3a	25441-25752	312	103	+1	Signal peptide (Secretory)	1-20	25427	**AACGAAC**(7) AUG
ORF3b	25667-25957	291	96	+2				
ORF4a	25709-25957	249	83	+2	Double stranded RNA binding domain<br>Type I Interferon Antagonist	3-73	25700	**AACGAAC**(4) AUG
ORF4b	25947-26624	678	225	+3	Nuclear Localization Signal (NLS)<br>Two 2′,5′-phosphodiesterase (PDE) motifs, AKAP18 Delta<br>Host RNase L activation inhibitor	25-50<br>105-108, 188-191		

ORF5	26634-27320	687	228	+3	Signal peptide (Secretory)<br>Three transmembrane domains	1-28<br>31-55, 67-87, 93-112	26626	**AACGAAC**(1) AUG
E	27395-27643	249	82	+2	Transmembrane domain	12-34	27387	**AACGAAC**(1) AUG
M	27654-28310	657	218	+3	Three Transmembrane domains	20-38, 50-71, 77-95	27642	**AACGAAC**(5) AUG
N	28365-29648	1284	427	+3	RNA Binding domain<br>Dimerization domain	20-171<br>243-361	28342	**AACGAAT**(16) AUG
ORF8b	28411-28977	567	188	+1	Unknown			

[a] ADRP, ADP-ribose 1 -phosphatase; PLPro, papain-like protease; 3CLpro, 3C-like protease; RdRp, RNA-dependent RNA polymerase; Hel, helicase; ExoN, 3′-to-5′ exonuclease; N7-MTase, (guanine-N7)-methyltransferase; NendoU, nidoviral uridylate-specific endoribonuclease; O-MT, 2′-O-ribose methyltransferase.

[b] Boldface indicates putative TRS sequences

Table S4. *Cleavage site used between nsps in members of Merbecovirus*

	Cleavage site							
**nsp**	**Ea-HedCoV HKU31**	**Erinaceus CoV**	**MERS-CoV**	**NeoCoV**	**SC2013**	**Ty-BatCoV HKU4**	**Pi-BatCoV HKU5**	**Hp-BatCoV HKU25**
nsp1/nsp2	G/D	G/D	G/D	G/D	G/D	G/D	G/D	G/D
nsp2/nsp3	G/A	G/A	G/A	G/A	G/A	G/M	G/A	G/A
nsp3/nsp4	G/S	**G/S**	G/A	G/A	G/A	G/A	G/A	G/A
nsp4/nsp5	Q/S	Q/S	Q/S	Q/S	Q/S	Q/S	Q/S	Q/S
nsp5/nsp6	Q/S	Q/S	Q/S	Q/S	Q/S	Q/S	Q/S	Q/S
nsp6/nsp7	Q/S	Q/S	Q/S	Q/S	Q/S	Q/S	Q/S	Q/S
nsp7/nsp8	**Q/S**	**Q/S**	Q/A	Q/A	Q/A	Q/A	Q/A	Q/A
nsp8/nsp9	Q/N	Q/N	Q/N	Q/N	Q/N	Q/N	Q/N	Q/N
nsp9/nsp10	Q/A	Q/A	Q/A	Q/A	Q/A	Q/A	Q/A	Q/A
nsp10/nsp12	H/S	H/S	Q/S	Q/S	Q/S	Q/S	Q/S	Q/S
nsp12/nsp13	Q/A	Q/A	Q/A	Q/A	Q/A	Q/A	Q/A	Q/A
nsp13/nsp14	Q/S	Q/S	Q/S	Q/S	Q/S	Q/S	Q/S	Q/S
nsp14/nsp15	Q/G	Q/G	Q/G	Q/G	Q/G	Q/G	Q/G	Q/G
nsp15/nsp16	**Q/G**	Q/A	Q/A	Q/A	Q/A	Q/A	Q/A	Q/A

Unique cleavage sites are in bold
